# Separation Techniques for Quantification of Radionuclides in Environmental Samples

**DOI:** 10.1100/tsw.2009.124

**Published:** 2009-11-01

**Authors:** Dusan Galanda, Pavol Rajec, Lubomir Mátel, Olga Rosskopfová, Silvia Dulanská

**Affiliations:** Department of Nuclear Chemistry, Faculty of Science, Comenius University, Bratislava, Slovakia

**Keywords:** radionuclides, plutonium, americium, strontium, uranium, extraction, nuclear chemistry

## Abstract

The reliable and quantitative measurement of radionuclides is important in order to determine environmental quality and radiation safety, and to monitor regulatory compliance. We examined soil samples from Podunajske Biskupice, near the city of Bratislava in the Slovak Republic, for the presence of several natural (U, Th, K) and anthropogenic (Cs, Sr, Pu, Pu, Am) radionuclides. The area is adjacent to a refinery and hazardous waste processing center, as well as the municipal incinerator plant, and so might possess an unusually high level of ecotoxic metals. We found that the levels of both naturally occurring and anthropogenic radionuclides fell within the expected ranges, indicating that these facilities pose no radiological threat to the local environment. During the course of our analysis, we modified existing techniques in order to allow us to handle the unusually large and complex samples that were needed to determine the levels of Pu, Pu, and Am activity. We also rated three commercial techniques for the separation of 90Sr from aqueous solutions and found that two of them, AnaLig Sr-01 and Empore Extraction Disks, were suitable for the quantitative and reliable separation of Sr, while the third, Sr-Spec Resin, was less so. The main criterion in evaluating these methods was the chemical recovery of Sr, which was less than we had expected. We also considered speed of separation and additional steps needed to prepare the sample for separation.

### INTRODUCTION

The measurement of radionuclides in the environment is important for radiation safety, for monitoring environmental quality, and for determining regulatory compliance. Inhabitants of areas adjacent to hazardous waste dumps and waste reprocessing areas have to be especially concerned about the emissions and regulatory compliance of these facilities, as noncompliance or accidental release could severely affect their environment. The area of Podunajske Biskupice in the city of Bratislava, capital of the Slovak Republic, is one such area. It is adjacent to the Slovnaft refinery, which also processes hazardous waste, and to the city's municipal waste incinerator. This area has been shown to have an extremely high level of Cu and elevated levels of Zn, Pb, As, and Cd[1], while the area surrounding the incinerator plant has been shown to have above-average levels of all these elements plus B, Mo, Ni, and Co. Presently, these elements are chemically bound to illite and kaolinite, to the iron and manganese oxides, and to some of the organic materials in the soil as a result of its alkaline character[1]. There is concern that a general drop in pH, arising, for example, from acid rain, might free these heavy metals from the matrix, leading to contamination of the groundwater. Naturally, the presence of radionuclides in this area, and their possible release into the water table, would generate concern and it would be vitally important to determine their presence and concentration.

Two main groups of radionuclides are usually subject to monitoring, natural ones and anthropogenic ones. Natural radionuclides include the alpha emitters U, Th, and their progeny. Some of these are also radiotoxic alpha emitters and a strict concentration limit of 0.1 mSv/year has been established for them[2]. Ra, Rn, and Po are the most important of the alpha emitters and their concentration in tapwater has been limited to less than 0.1 Bq/l, while their gross alpha activity cannot exceed 1.0 Bq/l[3]. While measurement of the gross alpha and beta activity remains the primary method for activity determination in water samples, an increasing number of laboratories have begun to measure individual radionuclides, such as $^{226}$Ra, $^{210}$Pb, $^{210}$Po, and natural U.

Anthropogenic radionuclides, including the alpha emitters $^{241}$Am, $^{239}$Np, and $^{239}$Pu, are produced during nuclear reactions. The nuclear power industry is permitted by health authorities to release small, controlled amounts of specific radionuclides on a continuous basis into the environment[4]. Larger amounts can be released by nuclear weapons tests, atmospheric satellite burn-up, or by nuclear power accidents. Pu might be found in environmental samples as a result of fallout from nuclear weapons tests, nuclear reactor accidents, discharge from reprocessing plants, or from leakage of radioactive waste.

We report here the determination of the presence and concentration of several radionuclides in soil samples taken from Podunajske Biskupice in the vicinity of the plants. Owing to the complex nature of the samples and to the low levels of Pu present, it was necessary for us to modify existing techniques[5,7, 8,9,10,11,12,13,14,15,16,17,18,19] in order to handle the relatively large amounts of complex samples. Radionuclide separation was carried out using Aliquat-336® (Cognis) according to standard procedures[30], but with a few modifications in order to enhance the recovery from large samples. For the initial extraction, we used 30% tricaprilamine nitrate (Aliquat-336 conditioned in nitric acid) in toluene rather than xylene. The radionuclides, except for Pu and U, were stripped from this reagent using a mixture of 8 *M* nitric acid with 0.5 *M* $NaNO_2$ and 0.35 *M* $H_2O_2$. U was stripped from the reagent using 8 *M* nitric acid alone and Pu was quantitatively recovered with 50 ml 0.025 *M* oxalic acid in 0.15 *M* HCl. Pu, U, and Am were coprecipitated with $NdCl_3$ rather than the more usual $CeF_3$. The analytical precision of the methods used is 93 ± 5% for $^{239}$Pu, 99 ± 10% for $^{238}$Pu, 96 ± 4% for $^{241}$Am, and 97 ± 11% for $^{234}$U (mean ± 1 sigma).

The apparatus used for beta particle measurement has an efficiency of 19.6 ± 0.6% and for alpha particle measurement, the average efficiency was 21 ± 0.5%.

The precision of the radiochemical methods is shown by the radiochemical recoveries of the tracers used. The recovery for Sr was in the range of 59.86–85.13%, with an average value of 72.50%.

For Pu, the recoveries were in the range 50.36–96.17%, with an average value of 71.20% and for Am, recoveries were in the range 34.94–84.54%, with an average value of 59.74%.

We are pleased to report that our techniques meet proper ISO17025:2005 standards. Since certification, a number of samples from different areas of the Czech and Slovak Republics have been sent to us for evaluation. We will also briefly describe their application to samples recently sent to us from the uranium-mining district of Novoveska Huta in Eastern Slovakia.

### METHODS

#### Sample Preparation

Soil samples were ground, homogenized, dried to a constant weight at 105°C for at least 8 h, and then ashed at 550°C. All methods used are standard for decay samples, which was verified in many experiments. Tracers ($^{242}Pu$, $^{243}Am$, $^{232}U$) and carriers ($Sr^{2+}$, $Y^{3+}$) were then added and the samples were digested with 8 *M* nitric acid and 30% $H_2O_2$. The digestion procedure varied depending on the size of the sample:

1. 20-g samples were mixed with 50 ml 8 *M* $HNO_3$ and 2.0 ml of 30% $H_2O_2$, and digested in an autoclave at 150°C for 8 h. The digestion was repeated twice.
2. 5-g samples were mixed with 20 ml 8 *M* $HNO_3$ and 3 ml 30% $H_2O_2$, and digested on a hot plate at 70°C for about 8 h. The digestion was repeated three times.
3. 0.5-g samples were mixed with 4 ml concentrated $HNO_3$ and 100 µl 30% $H_2O_2$, and were digested in a microwave (microwave laboratory system, Milestone Ethos) using a 20-min ramp to 190°C, followed by 20-min heating at constant temperature.

In all three procedures, both the solid and liquid phases were separated, cooled, and centrifuged.

### Separation Techniques

#### *Separation of Plutonium*

Pu was separated by liquid-liquid extraction with 30% Aliquat-336 in toluene[20,21]. Aliquat-336 was diluted in 30% toluene and converted to the nitrate form by equilibration with 4 *M* and 8 *M* $HNO_3$. To ensure the complete conversion of Pu(III) to Pu(IV), 2–3 g of sodium nitrite were added to the leaching before Pu separation[22].

The aqueous phase containing $^{241}Am$, $^{90}Y$, and $^{90}Sr$, which was left over after binding of Pu(IV) to Aliquat-336, was set aside for later analysis of $^{241}Am$ and $^{90}Sr$. Radionuclide impurities were washed out of the resin with 8 *M* $HNO_3$ (twice) and concentrated HCl (four times). Pu was eluted using 0.15 *M* HCl – 0.025 *M* $H_2C_2O_4 \cdot 2H_2O$. The eluate was evaporated to dryness and ashed in a muffle furnace for 30 min at 550°C. After cooling, the residue was dissolved in 4 ml of concentrated $HNO_3$ and evaporated to dryness followed by a similar treatment with concentrated HCl. Finally, the residue was dissolved in 6 ml 1 *M* HCl and a sample for alpha spectrometry was prepared.

#### *Separation of Americium*

The aqueous solution remaining after Pu separation was evaporated and the residue dissolved in a mixture of 0.1 *M* $HNO_3$/4 *M* $NaNO_3$. Am was separated by liquid-liquid extraction using 0.3 *M* TOPO/toluene[20] and eluted with 4 *M* $HNO_3$, followed by washing with toluene (twice). The solution was evaporated and the residue dissolved in 6 ml 1 *M* HCl. The final sample for alpha spectrometry was prepared by microcoprecipitation with $NdF_3$[20,23] (see following section).

#### *Activity Measurement of Plutonium and Americium*

Pu and Am were both coprecipitated from 1 *M* HCl, using 40% HF as a precipitant and Nd as a carrier[20,23]. The preparation of U source for measure was prepared by coprecipitation with $NdF_3$ also, but for coprecipitation of U, $TiCl_3$ must be added in order to reduce the oxidative state of U. After the precipitation had settled (30 min at –20°C), it was homogenized using ultrasonic equipment and the $NdF_3$ suspension was filtered through a 25-mm, 0.1-µm filter. The filter was dried and the activity of the actinides was determined using alpha spectrometry using Ultra™ ion-implanted silicon detectors with a 600-mm$^2$ active area (EG&G ORTEC, Model 676A).

### Separation of Uranium from both Aqueous and Soil Samples[24]

For aqueous samples, a $^{232}U$ tracer was added to a 1-l sample, followed by evaporation to dryness. The residue was dissolved in 10 ml 1 *M* $Al(NO_3)_3$ – 3 *M* $HNO_3$. A UTEVA® Resin (Eichrom) prepacked column in a vacuum box was conditioned with 5 ml of 3 *M* $HNO_3$, followed by introduction of the sample. The column was then washed two times with 5 ml 3 *M* $HNO_3$, and the sorbent was converted to the chloride form using 5 ml 9 *M* HCl and washed with 20 ml 0.05 $C_2H_2O_4$ in 5 *M* HCl to remove Pu, Np, and Th from the column. U was then eluted with 10 ml of 0.01 *M* HCl. The activity of U was measured using alpha spectrometry using Ultra™ ion-implanted silicon detectors with a 600-mm$^2$ active area (EG&G ORTEC, Model 676A).

For soil samples, the $^{232}U$ tracer was added to the sample, which was then digested as described for the 20-g sample above. Following digestion, the leaching solution was treated similarly as the aqueous sample above.

### Separation of Yttrium from Strontium

The strontium fraction was evaporated to dryness and the residue was dissolved in concentrated $HNO_3$. The concentration of $^{90}Sr$ was determined by beta counting the activity of its daughter $^{90}Y$, using the time between $^{90}Sr$ – $^{90}Y$ separation and the beginning of beta measurement to determine the decay correction of $^{90}Y$. For each experiment, the process time needed for separation of $^{90}Sr$ from $^{90}Y$ was measured exactly from the moment when $^{90}Sr$ had been completely separated from $^{90}Y$. During calculation, incorporated time of decay into the mathematical formula was used for determining the concentration. Y was separated by liquid-liquid extraction using tributyl phosphate (TBP), which had been pre-equilibrated with concentrated $HNO_3$[22] Samples were agitated for 5 min, and the organic and aqueous phases were separated; Y was in the organic phase and Am and Sr were left in the aqueous phase. The TBP was washed with concentrated $HNO_3$, followed by elution of Y with 15 ml of deionized water and 15 ml 2 *M* $HNO_3$. $^{90}Y$ was precipitated as $Y_2(C_2O_4)_3.9H_2O$ by the addition of 30 ml of saturated ammonium oxalate solution. The eluate was heated on the hot plate (70°C) for 15 min with occasional stirring, followed by cooling and recovery of the $Y_2(C_2O_4)_3.9H_2O$ by filtering (Whatman No. 42 filter paper). The count rate of $^{90}Y$ of beta particle was determined using a low-level alpha-beta counter Tesla NRR 610 and Tesla NA 6201. The yield of Y was determined from the ratio of the weight of Y oxalate precipitated to the expected weight of Y oxalate (as determined from the Y carrier standardization).

### Evaluation of Modern $^{90}Sr$ Separation Techniques

Standards consisting of 150 µl of $^{90}Sr$ in 2 *M* $HNO_3$ (activity 57 Bq/l) were spiked with $^{85}Sr$ as a tracer and diluted to a final volume of 30 ml. In the final elution step, the $^{90}Sr$-containing fraction was eluted into a Cerenkov counting vial. Recovery of $^{85}Sr$ was determined using an HPGE detector at the 514-keV line. The $^{90}Sr$ activity of the solution was measured by Cerenkov counting over a 2-week period for the ingrowth of $^{90}Y$ using a TRI CARB LSC counter.

AnaLig® Sr-01[25] (IBC Advanced Technologies) was used per the manufacturer's instructions. After sample loading, the column was washed with 1 *M* $HNO_3$. The time when the last rinse completely drained through each column was taken as the starting point for $^{90}Y$ ingrowth. Sr was eluted with 0.04 *M* $Na_4EDTA$ into a Cerenkov counting vial.

Empore™ Strontium Rad Disks (3M) were preconditioned with $CH_3OH$ and $HNO_3$ following the manufacturer's instructions. After loading the sample on the disk, it was washed with 2 *M* $HNO_3$. The end of this rinse was taken as the start of $^{90}Y$ ingrowth. Sr was eluted with 0.04 *M* $Na_4EDTA$ into a Cerenkov counting vial.

Sr-Spec Resin[26] (Eichrom) was prepared and preconditioned with 8 *M* $HNO_3$ following the manufacturer's instructions. After sample loading, the column was washed with 8 *M* $HNO_3$. As before, the end of this wash was taken as the starting point for $^{90}Y$ ingrowth. Sr was eluted with 0.05 *M* $HNO_3$ into a Cerenkov counting vial and activity measurement proceeded as described above.

### Reagents Used

In the work reported above, all radionuclide carriers and tracers except $^{232}U$ were supplied by Lacomed (Bratislava, Slovakia) and manufactured by the National Physical Laboratory. The carriers $^{242}Pu$ and $^{243}Am$ had a specific activity of 12.47 ± 0.1 Bq/g. $^{232}U$ was produced locally and had a specific activity of 5.3 ± 0.1 Bq/g. The tracer had been prepared by older tracer $^{232}U$ in 0.1 *M* $HNO_3$. In order to prepare the tracer for use, we cleaned it from decay product using process with UTEVA Resin by Eichrom[13]. After pollutants elimination, activity of tracer was determined and standardized using valid method of alpha spectrometry and mathematical methods. $^{85}Sr$ had a specific activity of 20.0 ± 0.1 Bq/g, while the $^{90}Sr$ used for the standards had an activity of 5.0 ± 0.1 Bq. Other reagents were obtained from Sigma-Aldrich and were of chemical grade purity. For spectroscopic measurements, the minimal detection activity (MDA) was estimated using the Currie criteria[27]. For alpha radiation, MDA was 0.0008 Bq, for beta it was 0.01 Bq, and for gamma it was 0.3 Bq.

## RESULTS AND DISCUSSION

The presence of a high concentration of radionuclides in soil or groundwater is of great ecological concern, particularly for countries like Slovakia that have a nuclear power industry. Likewise, the quantitative separation of particular radionuclides from complex environmental samples is of growing interest. The area of Podunajske Biskupice in Bratislava, the capital of the Slovak Republic, adjacent to both a hazardous waste processing facility and the municipal incineration plant, is a good example of the kinds of sites where such measurements are important. The radionuclides $^{137}Cs$, $^{90}Sr$, $^{241}Am$, $^{239}Pu$, and $^{240}Pu$ were found in samples taken from this area. (The specific activity of $^{238}Pu$ in these samples fell below the MDA cutoff, while technical difficulties prevented us from measuring $^{241}Pu$.) Because of the low Pu- and Am-specific activity of the samples, it was necessary to use unusually large sample sizes of 20 g. After digestion, Pu and Am were coprecipitated with $HF/NdF_3$ and their activities measured by alpha spectroscopy. $^{137}Cs$ and $^{40}K$ were measured using gamma spectroscopy directly, while $^{232}Th$ was measured using gamma spectroscopy indirectly through its decay products $^{212}Pb$, $^{228}Ac$, and $^{208}Tl$. $^{90}Sr$ was isolated using TBP extraction (it is important to note that $^{90}Sr$ was isolated only after the precipitation of Am). U was separated using UTEVA Resin. The results are shown in Table 1.

The levels of natural $^{40}K$ as well as those radionuclides arising from the decay series of U and Th are in accordance with the average concentration of these radionuclides in the dominant soil types of the Czech and Slovak Republics[28]. Figs. 1 and 2 show typical alpha spectra of the U and Th fractions of the samples. These spectra show that we have successfully separated U and Th from the remaining alpha emitters, and that the relative abundances of U and Th isotopes and daughter nuclides are what would be expected from a natural sample. No unusually high levels of U, Th, or their daughter radionuclides are present.

The presence of $^{239}Pu$, $^{240}Pu$, $^{241}Am$, and $^{137}Cs$, none of them naturally occurring isotopes, is most likely due to residual fallout from nuclear weapons tests and from the Chernobyl disaster of 1986. The cumulative effect of these events has been the deposition of a small, but measurable, level of these radionuclides in the soil of the Czech and Slovak Republics. As part of a general chemical survey of the local environment, the presence and levels of both natural and anthropogenic radionuclides were measured in the area of Podunajske Biskupice. Because elevated levels of heavy metals had previously been found in this area owing to its proximity to the Slovnaft industrial complex and the municipal waste

**TABLE 1**
**The Specific Activities (Bq/kg) of Soil Samples from the Area of Podunajske Biskupice**

| Radionuclide | Bq/kg |
|---|---|
| $^{137}Cs$ | 14.1–83.8 |
| $^{90}Sr$ | 3.8–29.2 |
| $^{239,240}Pu$ | 0.130–2.904 |
| $^{241}Am$ | 0.074–0.580 |
| $^{40}K$ | 481 ± 159 |
| $^{238}U$ | 27.3 ± 4.5 |
| $^{232}Th$ | 29.2 ± 4.6 |

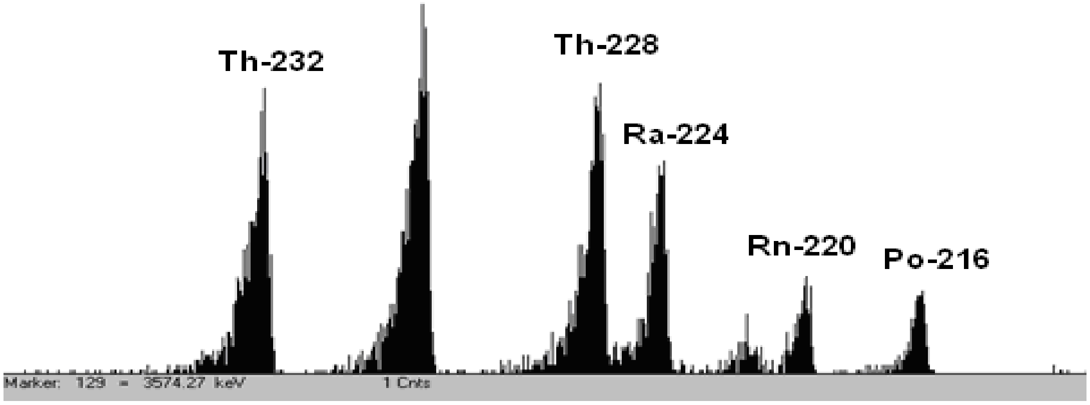

**FIGURE 1**. Alpha spectrum of Th fraction after separation soil sample.

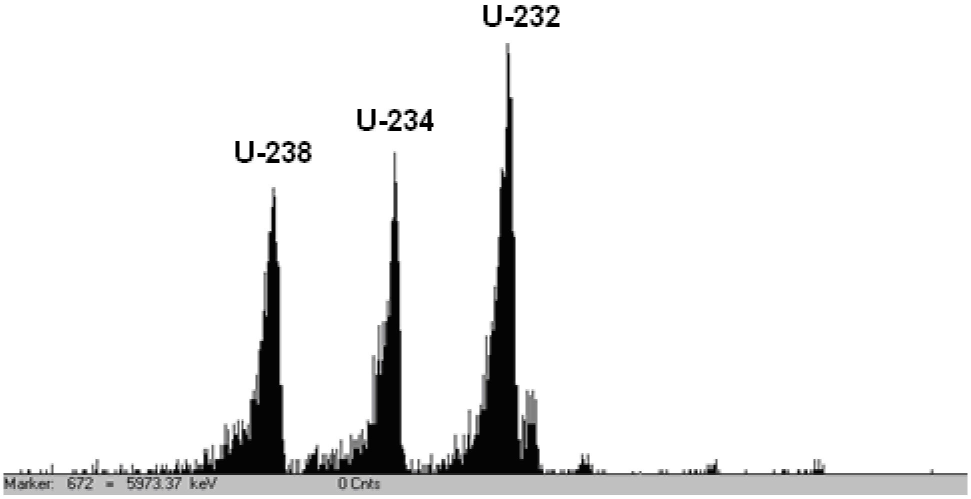

**FIGURE 2.** Alpha spectrum of U fraction after separation soil sample.

incineration plant[1], it seemed reasonable to suppose that any emissions of prohibited radionuclides from either facility might also be found here. The levels of $^{239}$Pu, $^{240}$Pu, $^{241}$Am, $^{137}$Cs, and $^{238}$U that we measured were, however, not unusual, falling within the range expected for this area. For example, average values of $^{239}$Pu concentration in Slovak soil are 0.32–3.11 Bq/kg, average values of $^{241}$Am are 0.03–0.63 Bq/kg, average values for $^{137}$Cs are 0.00–52 Bq/kg.

Radiation emissions from these two facilities are, therefore, unlikely to pose a menace to the environment.

### Uranium from Novoveska Huta

Normally, the activity ratio $^{234}$U/$^{238}$U <1 in aqueous samples. In 1992, Osmond and Ivanovich reported that in some samples they had encountered, this ratio was actually >1. They attributed this finding to different chemical behaviors for the two isotopes in water, although the source of this difference remains unclear[29]. In a series of mine water samples from the area of Novoveska Huta in East Slovakia, which were sent to us for analysis, we observed that for some samples, the activity ratio of $^{234}$U/$^{238}$U is also >1 (Fig. 3), allowing us to confirm the previous observations.

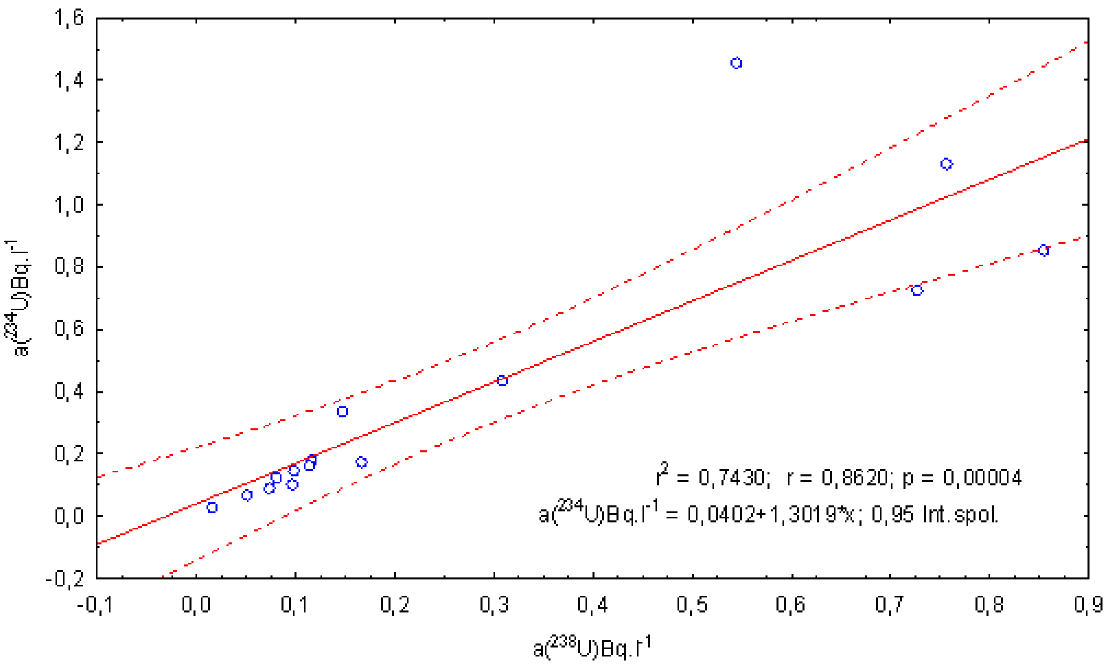

**FIGURE 3.** A correlation graph of $^{234}$U volume activity vs. $^{238}$U for the mine water samples collected in the areas Novoveska Huta of East Slovakia.

### $^{90}$Sr Separation

The measurement of $^{90}$Sr by the extraction of $^{90}$Y using TBP is a reliable, but tedious and somewhat inefficient method, and recent developments aimed at simplifying $^{90}$Sr extraction rely on either chromatography or solid phase extraction methods. Most of these modern methods require shorter analysis times and smaller amounts of chemical reagents than the TBP method. We determined the efficiency of three commercial techniques: Empore Extraction Disks (from 3M), Sr-Spec Resin (from

Eichrom), and AnaLig Sr-01 resin (from IBC Advanced Technologies). All methods were found to be suitable for the separation of $^{90}$Sr. The highest yields were found to be with AnaLig Sr-01 and the Empore Extraction Disks (which exceeded the typical yield of TBP by 13–14%); the Sr-Spec Resin worked less well, with a ~25% lower yield than the others (Table 2). The reason for this poor performance is unclear and under investigation.

**TABLE 2**
**Radiochemical Yield and Activity Determined of Model Solution - 59 Bq/l $^{90}$Sr (18 Samples)**

| Method | Yield (%) | A $^{90}$Sr (Bq/l) |
|---|---|---|
| AnaLig Sr | 89 ± 4 | 61.39 ± 2.16 |
| Empore Sr | 88 ± 5 | 59.52 ± 1.45 |
| Eichrom Sr Resin | 68 ± 15 | 60.04 ± 2.7 |

### CONCLUSION

We have determined the activity levels of $^{137}$Cs, $^{90}$Sr, $^{239}$Pu, $^{240}$Pu, $^{241}$Am, $^{40}$K, $^{238}$U, and $^{232}$Th from complex soil samples from Podunajske Biskupice, an area in Slovakia near a hazardous waste processing center and a municipal incinerator. The uncertainty of specific activity was estimated as combined uncertainty with k≈2 according to the following equation:

$$U(a) = k \cdot u(a)$$

where

$$u(a) = a \cdot \sqrt{\left[\frac{u(n_y)}{n_y}\right]^2 + \left[\frac{u(E)}{E}\right]^2 + \left[\frac{u(R)}{R}\right]^2 + \left[\frac{u(Q)}{Q}\right]^2}$$

$n_y$ = sample counting rate (/sec), $u(n_y)$ = sample counting rate deviation, E = measured efficiency, u(E) = deviation of measured efficiency, R = recovery of radiochemical analyses, u(R) = deviation of recovery, Q = sample weight (kg), u(Q) = uncertainty in sample weight.

We have found that the levels of the most important naturally occurring and anthropogenic radionuclides in the area are all within the ranges expected for this area, indicating that the municipal incinerator and waste reprocessing plant likely pose no radioactive environmental hazard. Furthermore, we were able to confirm an unusual observation made previously[26], viz. that the activity ratio $^{234}$U/$^{238}$U can be >1 for some aqueous samples. Finally, we experimentally verified that AnaLig Sr-01 as well as Empore Extraction Disks are very suitable for the quantitative and reliable separation of Sr from aqueous solutions.

### ACKNOWLEDGMENT

This work was supported by Grant No. APVV-20-007105 from the State Committee for Scientific Research, APVV Agency, Slovakia.
